# Laparoscopy vs. Laparotomy for Management of Postpartum Complications—A Retrospective Cohort Study

**DOI:** 10.3390/jcm15051982

**Published:** 2026-03-05

**Authors:** Liat Mor, Ohad Gluck, Amit Kreiner, Ram Kerner, Shimon Ginath, Ran Keidar, Ron Sagiv

**Affiliations:** 1 Department of Obstetrics & Gynecology, The Edith Wolfson Medical Center, Holon 5822012, Israel; 2 Grey Faculty of Medicine, Tel Aviv University, Tel Aviv 6997801, Israel

**Keywords:** minimally invasive techniques, postpartum surgeries, uterine rupture, maternal outcomes

## Abstract

**Background:** Postpartum complications requiring surgical intervention are challenging due to physiologic and anatomic changes. While laparotomy remains standard, laparoscopy is increasingly used. We compared outcomes of laparoscopic management of postpartum complications versus open management of postpartum complications. **Methods:** This retrospective cohort study included patients undergoing surgical intervention within three weeks postpartum at a single tertiary center between 2010 and 2023. Approach selection was primarily time-dependent, following an institutional practice change in 2020. Demographic, operative, and postoperative outcomes were compared. **Results:** Sixty-two participants with postpartum complications necessitating surgical intervention were included: 54 in the laparotomy group and 8 who underwent laparoscopy. Demographic characteristics were similar between groups. The main indication for laparoscopy was suspected uterine scar defects (*p* = 0.006), while laparotomy was obtained mainly in cases of suspected bleeding (*p* = 0.001). Both groups had comparable operative time, though the laparoscopy group had a shorter postoperative admission (*p* = 0.043). **Conclusions:** Laparoscopy is feasible for various postpartum complications. It offers comparable operative times to laparotomy with shorter postoperative admissions. Therefore, it is a promising alternative in selected cases when surgical expertise is available.

## 1. Introduction

The postpartum period is characterized by a drastic transition in physiological parameters, including profound changes in maternal hemodynamic state and organ function [1,2]. Following childbirth, the maternal cardiovascular system undergoes a complex rebalancing act, transitioning from the hyperdynamic state of pregnancy to a gradual return to pre-pregnancy baseline [1,3]. Concurrently, the abdomen undergoes anatomical adjustments, with the uterus involuting and abdominal musculature adapting to the postpartum state [4,5]. These intricate hemodynamic and anatomical fluctuations present a unique challenge in the context of postpartum surgeries, requiring meticulous consideration by healthcare providers.

Postpartum complications requiring surgical exploration are uncommon but potentially life-threatening. Reported indications include uterine rupture or dehiscence, intra-abdominal hemorrhage, infected hematoma, and uncontrolled postpartum bleeding [6,7,8]. Although the overall incidence of re-laparotomy following delivery is low, estimated at approximately 0.2–0.7%, such events are associated with substantial maternal morbidity, prolonged hospitalization, blood transfusion, intensive care unit (ICU) admission, and in rare cases, mortality [9]. Importantly, the rising global cesarean section rate has increased the population at risk for uterine scar-related complications during the puerperium, further emphasizing the need for optimal surgical strategies in this setting.

Traditionally, laparotomy has been considered the standard surgical approach for postpartum complications. The rationale for open surgery includes rapid abdominal access, tactile feedback, perceived improved control of bleeding, and familiarity in urgent obstetric scenarios. In hemodynamically unstable patients, laparotomy has long been viewed as the safest and fastest method to achieve definitive source control. However, laparotomy is also associated with well-recognized drawbacks, including increased postoperative pain, higher rates of wound complications, and longer recovery times. As laparoscopic skills and techniques gradually improve, the laparoscopic handling of such cases is occasionally preferred due to its facilitation of superior visualization and shorter recovery times [10,11].

Despite these advantages, the application of laparoscopy in the immediate postpartum period remains controversial. Concerns include distorted anatomy due to uterine enlargement, hypervascularization, presence of blood clots or adhesions, and potential hemodynamic instability. Evidence supporting laparoscopic management in this setting is limited and primarily derived from small case series and reports. Previous studies have compared maternal and operative outcomes of laparoscopy vs. laparotomy in the management of postpartum uterine rupture, reporting a reduced need for blood products or intensive care unit (ICU) admissions in the laparoscopy group, shorter hospitalization times, and increased patient satisfaction [12,13], yet more studies are required to assess the safety and efficacy of laparoscopy in various postpartum complications.

In 2020, our institution implemented a practice shift, favoring laparoscopy as the primary approach for postpartum abdominal surgical interventions, regardless of the specific indication. This transition provided an opportunity to evaluate outcomes associated with minimally invasive surgery in the postpartum population and to compare them with outcomes from the preceding laparotomy-dominant era.

Therefore, the present study aimed to compare laparoscopic management versus open surgical management of various postpartum complications within three weeks of delivery. Specifically, we sought to evaluate operative characteristics, intraoperative findings, and postoperative outcomes, as well as to explore whether laparoscopy may offer clinical advantages over laparotomy in selected postpartum cases.

## 2. Methods

In this retrospective cohort study, we collected data from all cases of postpartum complications necessitating surgical interventions from a single tertiary medical center between 2010 and 2023. Patients included were in the immediate and sub-acute postpartum phase—up to three weeks postpartum—following a vaginal delivery, vacuum-assisted delivery, or a cesarean section. Permission to conduct this study was granted by the institutional ethics committee, and the need for informed consent was waived due to retrospective design (approval number 0045-23-WOMC).

Postpartum complications that necessitated surgical intervention included any of the following:

1. Uterine rupture suspected clinically with or without supporting sonographic findings based on localized pain, subinvolution, or persistent bleeding in prior CS patient.
2. Intraabdominal bleeding—hemodynamic instability and/or hemoglobin drop with imaging evidence (free fluid/hematoma).
3. Infection—sepsis or signs of infection requiring source control measurements following unsatisfactory response to conservative management (clinical signs include fever, uterine tenderness, rising inflammatory markers, and purulent discharge).

All aforementioned surgical interventions were handled via laparotomy until 2020. Afterward, laparoscopy became the recommended approach for all patients regardless of indication or time interval from delivery. All patients were counseled preoperatively regarding the available surgical options, potential benefits and risks of each approach, and possible conversion from laparoscopy to laparotomy. Written informed consent was obtained prior to surgery. Notably, all procedures were performed by senior gynecologic surgeons.

Laparotomy was performed using a Pfannenstiel incision both for post-cesarean section where the same incision was used and for patients necessitating laparotomy following a vaginal delivery. The abdominal layers were opened in a standard fashion, followed by systematic inspection of the abdominal cavity, uterus, and surrounding structures. Hemostasis was achieved as required, and definitive management, such as repair of uterine scar defects, evacuation of hematomas, drainage of infected collections, adhesiolysis, or control of active bleeding, was performed according to intraoperative findings.

For cases managed laparoscopically, a laparotomy setup was kept immediately available in the operating room to allow prompt conversion if necessary. The laparoscopic approach began with diagnostic laparoscopy to evaluate the source of pathology, followed by targeted intervention. Depending on intraoperative findings, this included evacuation of blood clots, identification and control of bleeding sources using energy devices or suturing techniques, drainage of infected collections, adhesiolysis, and repair of uterine scar defects. When uterine repair was required, tissue was debrided as needed, the bladder was reflected when indicated, and the myometrial defect was closed using one or more layers of absorbable sutures at the discretion of the operating surgeon.

Bottom of Form

Conversion to laparotomy was performed if adequate visualization or hemostasis could not be achieved safely.

We included all patients for whom we had access to full medical files describing delivery characteristics, postpartum course, surgical files, and postoperative admission follow-up. Excluded were patients with missing data, surgical interventions not including the abdominal cavity, and cases with complications requiring non-gynecological repair.

The data collected included the demographic and delivery characteristics of the patients, maternity ward admission information, and elaboration of course of complication before surgical repair. The outcomes assessed encompassed operative and postoperative characteristics, including operative time and subjective intraoperative measures—such as presence of adhesions and signs of infection, as reported by the lead surgeon; postoperative admission time; need for blood products; need for an additional surgical intervention; and ICU admission.

The prevalence of the mentioned characteristics and outcomes was compared between the study groups using Student’s T-test or Mann–Whitney U-test for continuous data. Categorical data was analyzed using the chi-square test or Fisher’s exact test as appropriate. To account for the small sample size, we conducted effect size analyses using Cohen’s test, where a result of d = 0.2–0.5 is considered a small effect, a result of d = 0.5–0.8 is considered a medium effect, and a result greater than 0.8 is considered a large effect. All tests were two-sided, with statistical significance defined as *p* < 0.05. Analyses were considered exploratory due to the limited sample size and multiple comparisons. Therefore, no formal adjustment for multiplicity was applied.

## 3. Results

During the study period, a total of 48,280 deliveries occurred in our institution, of them 73 postpartum patients presented with complications requiring surgical interventions (1.5%). Eleven patients were excluded—five due to above-fascia interventions and six that were solely operated by non-gynecologists due to other organ damage. The remaining 62 patients included 54 patients who underwent laparotomy and 8 patients treated by laparoscopy.

Table 1 presents the demographics and pregnancy characteristics of the study groups. Notably, no significant differences were demonstrated between the demographics of the two groups. Regarding labor characteristics, the laparoscopy group had significantly higher rates of vaginal delivery in the delivery preceding the complication and accordingly lower rates of cesarean section (37.5% vs. 5.5%, *p* = 0.024 and 50% vs. 92.5%, *p* = 0.007, respectively, d = 1.14, indicating a large effect). Other delivery traits were similar between the groups.

Table 2 demonstrates the immediate postpartum outcomes of the groups before the surgical intervention. Higher rates of patients in the laparotomy group required blood products before surgical intervention (48.1% vs. 0%, *p* = 0.016, d = 1.01, indicating a large effect). Additionally, the suspected reason requiring surgical intervention according to clinical, laboratory, and imaging evaluation is detailed. Patients in the laparoscopy group had higher rates of suspected uterine scar defect but lower rates of suspected active bleeding compared to patients on the laparotomy group (37.5% vs. 3.7%, *p* = 0.006, and 12.5% vs. 66.6%, *p* = 0.001, respectively, d = 1.63 and d = 1.32, both indicating large effects).

The postpartum surgical intervention course and outcomes are presented in Table 3. No significant difference was noted with regard to operative time. During the surgical intervention, higher rates of adhesions and uterine scar defects were demonstrated in the laparoscopy group (62.5% vs. 12.9%, *p* = 0.005, for both, d = 1.35 for both, indicating large effects). Additionally, the laparoscopy group had a higher rate of signs of contamination and infection (62.5% vs. 22.2%, *p* = 0.030, d = 0.93, indicating a large effect). There were higher rates of uterine scar repair in the laparoscopy group (62.5% vs. 12.9%, *p* = 0.005, d = 1.35, indicating a large effect).

Table 4 presents the postoperative outcomes of the study groups. Notably, the laparoscopy group had a significantly shorter postoperative admission time (median = 3 IQR 2.25–6 vs. median = 6.5 IQR 5–9, *p* = 0.043, d = 1.04, indicating a large effect). No other significant differences were demonstrated.

A detailed elaboration of all laparoscopy cases is presented in Supplementary Table S1.

## 4. Discussion

This study aimed to compare the outcomes and complications of laparoscopy vs. laparotomy as the primary surgical intervention in various postpartum complications. The main outcomes were as follows: (1) Laparoscopy is a feasible and effective tool for various postpartum complications. (2) Laparoscopy by trained physicians does not increase the operative time compared to laparotomy and requires shorter postoperative admission. (3) In this cohort, laparoscopy was performed in sub-acute cases presenting symptoms raising suspicion for uterine scar defect and active infection, while laparotomy was performed mostly in cases of active bleeding. The utilization of laparoscopy in more acute cases should be investigated further.

Postpartum complications requiring surgical interventions are rare, with inconsistent rates described in the literature, ranging between 0.003 and 0.7% [14,15,16,17]. A previous study by Witteveen demonstrated an increased risk for such complications in patients undergoing a CS or following a vaginal birth after CS (VBAC) compared to patients after a vaginal delivery with an unscarred uterus [18]. Most studies to date have presented rates of relaparotomy following a cesarean section with scarce information regarding the incidence of postpartum complications necessitating surgical interventions following vaginal deliveries, with most data relevant to this issue being retrieved from case reports [19,20]. Our study reports a rate of 0.15% of postpartum surgical interventions, similar to the rates reported above, with higher incidence among patients in the post CS subgroup.

Ashwal et al. reported that the indication for postpartum surgical intervention varies according to the time elapsed since delivery [15]. In their study, procedures performed in the immediate postpartum period were most commonly prompted by acute hemorrhage, whereas surgeries undertaken later in the puerperium were more frequently related to infectious complications or uterine scar dehiscence. This temporal pattern likely reflects the natural evolution of postpartum pathology, with bleeding presenting abruptly and requiring urgent intervention, while infectious processes or scar-related defects tend to develop more gradually. In our cohort, a similar pattern was suggested. Though not statistically significant, there was a trend toward a longer interval between delivery and surgical intervention in the laparoscopy group compared with the laparotomy group. This observation may partially reflect the distribution of indications, as laparoscopic cases more often involved suspected scar defects or infection, conditions that typically manifest in the sub-acute postpartum phase. Conversely, laparotomy was more commonly performed in cases of suspected active bleeding, which generally presents earlier and may necessitate more immediate operative management.

Though most cases in the laparotomy group were in an acute setting with different indications for surgical intervention compared to the laparoscopy group, this divergence does not reflect a selective clinical preference. As stated above, an institutional shift in surgical practice from 2020 recommended all postpartum cases to be handled via a laparoscopic-first policy, irrespective of symptom duration or presumed etiology. Thus, the choice of surgical approach was largely determined by calendar period rather than clinical stratification.

The use of laparoscopy in hemodynamically unstable patients remains a subject of ongoing debate. Traditionally, open laparotomy has been considered the preferred approach in unstable cases due to the perceived need for rapid abdominal access and immediate hemorrhage control. Concerns regarding potential delays in management have historically limited the adoption of minimally invasive techniques in acute obstetric and gynecological emergencies. However, accumulating evidence suggests that, in experienced hands, laparoscopy can be safely performed even in unstable patients without compromising outcome [21,22]. A previous study from our group demonstrated favorable outcomes using laparoscopy in acute settings involving hemodynamically unstable patients [23]. Consistent with that experience, early laparoscopic interventions were also performed in the present cohort, including cases undertaken as early as 8 h postpartum and in patients presenting with clinical instability. These observations support the growing body of evidence indicating that minimally invasive surgery may be feasible across a broad range of clinical scenarios, provided that appropriate surgical expertise and readiness for conversion to laparotomy are ensured.

However, the utility of laparoscopy for varied postpartum indications is still to be proven. Several small studies have demonstrated non-inferiority of laparoscopy for the treatment of uterine rupture compared to the open approach [12,13], and the advantages of laparoscopic treatment in cases of postpartum infection, bleeding, or drainage of hematomas have only thus far been described in case reports [11,24,25,26,27]. This study affirms the efficacy of laparoscopy for diverse postpartum complications, suitable for different indications, with no added complications. However, transition from a laparoscopic to an open approach was required in one case within this cohort due to gross adhesions and blood clots obscuring visualization and interrupting the understanding of anatomy.

No studies to date discussed the optimal timing of laparoscopic intervention postpartum. Our data demonstrates that early laparoscopy is significantly advantageous in cases of uterine scar defect and intraabdominal hematomas, reducing related morbidity, including peritonitis and infection, which were observed solely in late-treated cases compared to cases of earlier surgical interventions.

Although patient-reported outcomes were not formally assessed in this study, prior studies in the literature consistently demonstrate lower postoperative pain scores and higher patient satisfaction following laparoscopic compared with open surgery [13]. In our cohort, the significantly shorter postoperative admission time observed in the laparoscopy group may indirectly reflect these well-established benefits. Reduced tissue trauma, smaller incisions, and earlier mobilization are typically associated with decreased analgesic requirements and faster functional recovery, which may facilitate earlier discharge. In the postpartum context, diminished pain and shorter hospitalization are particularly meaningful, as they may support earlier ambulation, improved breastfeeding initiation, enhanced maternal–infant bonding, and overall maternal well-being. Future prospective studies incorporating validated pain and satisfaction measures are warranted to better elucidate these patient-centered advantages.

This study has a few notable strengths. First, it is the first study to explore the utility and efficacy of laparoscopy vs. laparotomy in various complex postpartum complications. Second, considering the rarity of these cases, this study presents a diverse cohort emphasizing the utility of laparoscopy in several scenarios. Lastly, since this study was a single-center study, we had access to all pregnancy, delivery, and postpartum data, allowing for meticulous case-by-case follow-up and an extensive understanding of the outcomes.

The limitations of this study include its retrospective design and the paucity of acute cases in the laparoscopy group—necessitating further, preferably larger, and randomized trials to appreciate the yield of laparoscopic handling in these cases. Second, the fact that the surgical approach differed across the study period might introduce a temporal bias, and refinement of operative skills over time may have influenced operative metrics. However, no other major differences were observed in baseline patient demographics, surgical team composition, or perioperative protocols during the study period, which may mitigate, though not eliminate, the magnitude of this temporal confounding. Moreover, since the indications for surgical interventions differed between the groups and the small sample size, which makes subgroup analyses not feasible, more data is required to affirm the effectiveness of laparoscopy for all indications. Given the differences in clinical presentation, surgical outcomes may be influenced largely by variable indications rather than as a direct causal effect of surgical technique. Lastly, the size of the laparoscopic group, although fairly large compared to other studies, is small, meaning larger, perhaps multicenter, studies are required. Moreover, due to the small sample size, this study’s findings should be interpreted as exploratory.

## 5. Conclusions

Postpartum complications requiring surgical interventions are rare events, yet they are associated with increased maternal morbidity. Laparoscopic intervention in such cases is a feasible approach, with similar operative time to open surgeries, no added complications, and reduced postoperative admission time. Therefore, minimally invasive procedures may be a promising alternative in selected cases when surgical expertise is available. Future prospective, multicenter studies are needed to confirm these findings and better define the role of laparoscopy across different clinical settings.

## Figures and Tables

**Table 1:** Maternal demographics and pregnancy characteristics.

	Laparoscopy (*n* = 8)	Laparotomy (*n* = 54)	*p*-Value
Maternal age (years)	31.7 ± 4.8	32.7 ± 6.0	0.697
Nulliparity (%)	1 (12.5)	23 (42.5)	0.136
GA at delivery (weeks)	39.6 [37.4–40.8]	38.1 [36.7–39.6]	0.161
Previous cesarean section	7 (87.5)	26 (48.1)	0.055
BMI (kg/m^2^)	22.1 [22.0–24.2]	25.0 [21.5–31.6]	0.334
Pregestational & gestational diabetes (%)	1 (12.5)	5 (9.1)	0.772
Chronic hypertension (%)	0	1 (1.8)	1.0
Preeclampsia (%)	0	9 (16.6)	0.609
Smoking (%)	0	2 (3.7)	1.0
Thrombophilia (%)	0	2 (3.7)	0.580
Delivery characteristics			
Vaginal delivery (%)	3 (37.5)	3 (5.5)	**0.024**
Vacuum extraction (%)	1 (12.5)	1 (1.8)	0.243
Cesarean section (%)	4 (50.0)	50 (92.5)	**0.007**
Emergent cesarean section	1 (12.5)	17 (31.4)	0.418
Second-stage cesarean section	1 (12.5)	8 (14.8)	1.0
Adhesions during cesarean section	2 (25)	8 (14.8)	0.604
Intrapartum fever	1 (12.5)	2 (3.7)	0.344

Continuous variables are presented as mean ± SD or median [interquartile range]; categorical variables are presented as *n* (%). *p*-values in bold are statistically significant. GA—gestational age; BMI—body mass index (kg/m^2^).

**Table 2:** Postpartum outcomes of patients with subsequent complications before secondary surgical intervention.

	Laparoscopy (*n* = 8)	Laparotomy (*n* = 54)	*p*-Value
Time from birth to postpartum surgical intervention (days)	9.0 [1.6–16.7]	1.3 [0.4–8.2]	0.093
Blood products before surgical intervention	0	26 (48.1)	**0.016**
ICU before surgical intervention	0	3 (5.5)	1.0
Hemoglobin before surgical intervention	9.9 [7.6–12.1]	9.0 [7.5–10.7]	0.448
Leukocytes before surgical intervention	12.9 [8.8–17.0]	12.6 [9.8–16.7]	0.971
Postpartum surgical intervention reason			
Suspected uterine scar defect	3 (37.5)	2 (3.7)	**0.006**
Suspected infection	4 (50.0)	16 (29.6)	0.672
Suspected bleeding	1 (12.5)	36 (66.6)	**0.001**

Continuous variables are presented as mean ± SD or median [interquartile range]; categorical variables are presented as *n* (%). *p*-values in bold are statistically significant. ICU—intensive care unit. Several findings in the same patient have been reported separately.

**Table 3:** Outcomes of surgical interventions for various postpartum complications in patients undergoing laparotomy vs. laparoscopy.

	Laparoscopy (*n* = 8)	Laparotomy (*n* = 54)	*p*-Value
Operative time (minutes)	79 [44–87]	52.5 [40.7–72.7]	0.370
Conversion from laparoscopy to laparotomy	1 (12.5)	0	1.0
Intraoperative findings			
Adhesions	5 (62.5)	7 (12.9)	**0.005**
Active bleeding source	0	18 (33.3)	0.092
Oozing from small vessels	0	7 (12.9)	0.580
Blood clots, no bleeding source	1 (12.5)	13 (24.0)	0.670
Atonic uterus	0	3 (5.5)	1.0
Uterine scar defect	5 (62.5)	7 (12.9)	**0.005**
Signs of infection	5 (62.5)	12 (22.2)	**0.030**
No abnormal finding	0	3 (5.5)	1.0
Main interventions during postpartum surgery			
Adhesiolysis and pus drainage	2 (25.0)	14 (25.9)	1.0
Hemostasis of bleeding	1 (12.5)	33 (61.1)	0.069
Repair of uterine scar	5 (62.5)	7 (12.9)	**0.005**

Continuous variables are presented as mean ± SD or median [interquartile range]; categorical variables are presented as *n* (%). *p*-values in bold are statistically significant. Several findings in the same patient have been reported separately.

**Table 4:** Postoperative outcomes of patients following laparotomy vs. laparoscopy for various postpartum complications.

	Laparoscopy (*n* = 8)	Laparotomy (*n* = 54)	*p*-Value
ICU admission	1 (12.5)	14 (25.9)	0.666
Blood products after surgery	2 (25.0)	26 (48.1)	0.522
Postoperative admission time (days)	3 [2.25–6]	6.5 [5–9]	**0.043**
Relaparotomy/ re-laparoscopy	1 (12.5)	4 (7.4)	0.206

Continuous variables are presented as mean ± SD or median [interquartile range]; categorical variables are presented as *n* (%). *p*-values in bold are statistically significant. ICU—intensive care unit.

## Data Availability

The original contributions presented in this study are included in the article/Supplementary Material. Further inquiries can be directed to the corresponding author(s).

## Supplementary Materials

The following supporting information can be downloaded at: https://www.mdpi.com/article/10.3390/jcm15051982/s1, Table S1: Confirming data.
